# Neuropathic Pain as Main Manifestation of *POLG*-Related Disease: A Case Report

**DOI:** 10.3389/fneur.2022.846110

**Published:** 2022-03-08

**Authors:** Melanie Lang-Orsini, Paloma Gonzalez-Perez

**Affiliations:** ^1^Department of Pathology, Massachusetts General Hospital, Harvard Medical School, Boston, MA, United States; ^2^Department of Neurology, Massachusetts General Hospital, Harvard Medical School, Boston, MA, United States

**Keywords:** CPEO, polyneuropathy, neuropathic pain, POLG, mitochondrial disease

## Abstract

Mutations in nuclear-encoded genes that are involved in mitochondrial DNA replication and maintenance (e.g., *POLG*) have been associated with chronic progressive external ophthalmoplegia (CPEO) phenotype. These nuclear genome mutations may lead to multiple mitochondrial DNA deletions or mitochondrial DNA depletion. On the other hand, primary genetic defects of mitochondrial DNA (such as single large-scale deletion or point mutations) have also been associated with the CPEO phenotype. Chronic progressive external ophthalmoplegia (CPEO) may be a manifestation of specific syndromes that, when clinically recognized, prompt clinicians to investigate specific genetic defects. Thus, CPEO, as part of Kearns Sayre syndrome, suggests the presence of a large-scale deletion of mitochondrial DNA. However, in pure CPEO or CPEO plus phenotypes, it is more difficult to know whether causative genetic defects affect the nuclear or mitochondrial DNA. Here, we present a patient with a long-standing history of CPEO plus phenotype, in whom the sequencing of mitochondrial DNA from skeletal muscle was normal, and no other genetic defect was suspected at first. At the time of our evaluation, the presence of polyneuropathy and neuropathic pain prompted us to investigate nuclear genetic defects and, specifically, mutations in the *POLG* gene. Thus, the sequencing of the *POLG* gene revealed p.Thr251Ile and p.Pro587Leu mutations in one allele, and p.Ala467Thr mutation in another allele. Although one would expect that mutations in *POLG* lead to multiple mitochondrial DNA deletions or depletion (loss of copies), the absence of mitochondrial DNA abnormalities in tissue may be explained by heteroplasmy, a lack or no significant involvement of biopsied tissue, or a sampling bias. So, the absence of secondary mitochondrial DNA alterations should not discourage clinicians from further investigating mutations in nuclear-encoded genes. Lastly, mitochondrial point mutations and single mitochondrial DNA deletions very rarely cause CPEO associated with polyneuropathy and neuropathic pain, and POLG-related disease should be considered in this scenario, instead.

## Introduction

The human DNA polymerase gamma is formed by a 140 kDa catalytic subunit called POLG and a 55 kDa dimeric accessory subunit called POLG2. The POLG is encoded by the *POLG* gene (Chr.15q25) and the POLG2 is encoded by the *POLG2* gene (Chr.17q24.1). The POLG has DNA polymerase, 3′ to 5′ exonuclease, and 5′-deoxyribose activities, whereas POLG2 increases the affinity of POLG for mitochondrial DNA. Thus, the DNA polymerase gamma is entirely encoded by nuclear genes, although its main role is the replication of mitochondrial DNA. The *POLG* and *POLG2* mutations may lead to multiple deletions or depletion (loss of copies) of mitochondrial DNA (mtDNA) and have been associated with a broad spectrum of phenotypes such as chronic progressive external ophthalmoplegia (CPEO) (1).

CPEO is a slow, progressive, and painless ocular myopathy characterized by bilateral ptosis and limitation of ocular movements in all directions that is not usually associated with diplopia because, as in other ocular myopathies, no significant misalignment occurs between both eyes. Although other myopathies, such as oculopharyngeal muscular dystrophy, oculopharyngeal distal myopathy, or MHY2-myopathy, are also characterized by progressive ptosis with or without ophthalmoplegia, the term CPEO usually refers to mitochondrial ocular myopathies due to either mutation in mitochondrial or nuclear DNA. The CPEO may occur in isolation (pure CPEO), as part of specific syndromes, or associated with a constellation of clinical manifestations that have not been recognized as syndrome or disorder (CPEO plus). Thus, CPEO is a characteristic feature of Kearns Sayre syndrome (KSS) that is caused by a large-scale 1.1 to 10 kilobase deletion of mtDNA, and it can also be seen as a clinical manifestation of dominantly or recessively inherited *POLG*-related disease; mutations in this nuclear gene account for 25% of patients with CPEO phenotype (2–6).

Here, we report a patient who presented to us for evaluation of polyneuropathy and neuropathic pain as main symptoms, which were previously thought to be unrelated to her long-standing ocular myopathy for which no specific etiology was initially found. Although polyneuropathies are frequent (and commonly idiopathic in the absence of diabetes), they can be a manifestation of a mitochondrial disorder. Furthermore, polyneuropathy has been reported as a predictor of nuclear gene defects in patients with CPEO, and more specifically, the presence of neuropathic pain should prompt the clinician to consider *POLG*-related disease as an unifying diagnosis (7, 8).

## Case Description

A 69-year-old woman was referred to us for paresthesia, neuropathic pain, and cramps in extremities as her main symptoms. She was in good health until her early 50s when she first experienced slowly progressive bilateral ptosis that was surgically corrected twice, and restriction of eye movements in all directions that did not bother her much; she never experienced any diplopia. Eventually, she developed myalgias and fatigue. However, her main complaints were numbness and burning pain in her hands and feet that worsened over time, as well as cramps in her feet. She reported sensitivity to temperature; cold exacerbated burning pain in her feet mostly at night. She also felt clumsy; she frequently dropped things from her hands despite bilateral carpal tunnel release in the past, she had developed mild action hand tremors, and she often suffered near-falls because her balance had deteriorated over time. She was taking duloxetine 30 mg/day and pregabalin 300 mg/day with partial benefit of her burning pain, and she tried lidocaine patches on feet that did not provide any relief. She also reported a long-standing history of dysphagia to solids, with an isolated episode of aspiration in the past and episodes of retrosternal spasms during meals. She denied speech or chewing difficulties. She denied shortness of breath, hearing difficulties, cataracts, or heart problems. Her past medical history also included sleep apnea and cervical and lumbar spine surgeries. She denied alcohol or illicit drug use. Her older brother had similar ocular symptoms and carried a diagnosis of neuropathy; both of uncertain etiology too.

**Figure 1:**
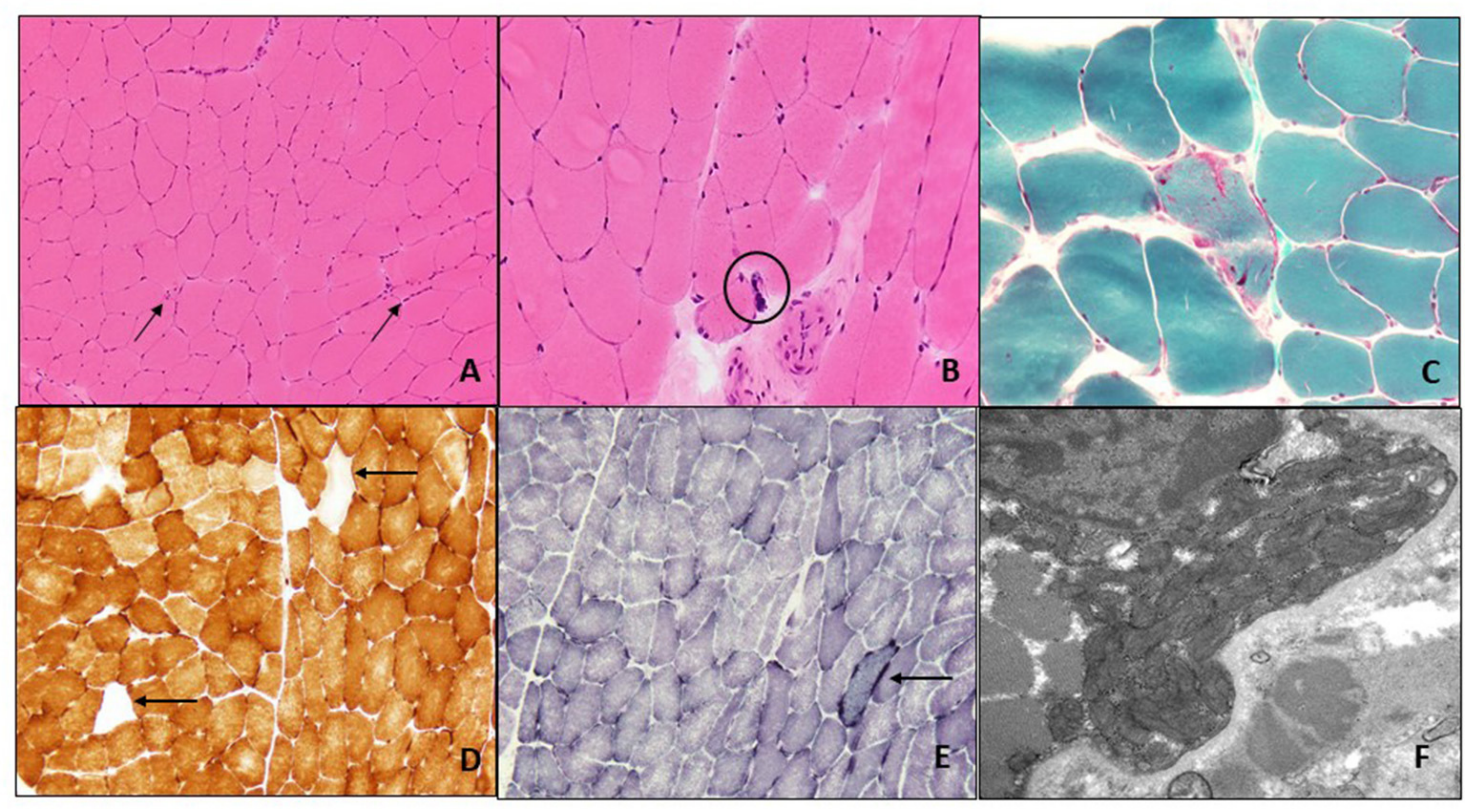
Muscle biopsy of left quadriceps: H&E-stained frozen section of the left quadriceps muscle biopsy (200X) shows occasional angulated atrophic fibers (arrows) and scattered internal nuclei **(A)**. At high power (400X) occasional nuclear bags are seen (circle) **(B)**. Gomori trichrome stain (400X) reveals subsarcolemmal accumulation of mitochondria with an irregular accumulation of Gomori-positive sarcoplasmic material **(C)**. There are scattered COX-negative fibers (arrows) **(D)**, and subsarcolemmal accumulation of mitochondrial is again seen on SDH staining (arrow) **(E)** (both 200X). Electron microscopy (22,000X) shows enlarged and pleomorphic mitochondria that were present in the subsarcolemmal region only **(F)**.

Before the first visit with us, she had undergone several tests since the symptom onset that we have summarized here. Thus, her serum creatine kinase was mildly elevated (268 U/L, ref: 33–211) and her baseline lactate was high (=5.5 nmol/L, ref: 0.5–2.2). Blood cell count, electrolytes, vitamin B12, serum protein electrophoresis, hemoglobin A1C, thyroid-stimulating hormone, C-reactive protein and erythrocyte sedimentation rate, carnitine and acylcarnitine levels, antinuclear antibodies, and acetylcholine receptor binding antibodies were all normal or negative. Urine organic acids were also normal. A brain CT scan did not show any intracranial abnormality. She underwent genetic testing for oculopharyngeal muscular dystrophy that was negative. She had two electrodiagnostic studies; both showed a length-dependent, axonal, and sensory polyneuropathy (sensory nerve action potentials of both sural nerves were absent, and sensory nerve action potentials of ulnar and radial nerves demonstrated reduced amplitudes and normal peak latencies). At the age of 59, she underwent a muscle biopsy of left quadriceps muscle that showed mild myopathic and neuropathic features; the former included occasional subsarcolemmal accumulation of mitochondria and scattered COX-negative muscle fibers, while the latter; angulated fibers, nuclear clumps, and mild fiber type grouping that were attributed to her history of lumbosacral radiculopathy (Figure 1). Although such mild mitochondrial findings within the sixth decade of life could be a consequence of normal aging, a manifestation of a mitochondrial disorder was also plausible. Sequencing of mtDNA from muscle tissue did not detect any point mutation or deletions. Furthermore, mitochondrial carnitine and CoQ10 levels, and activity of electron transport chain complexes were all normal from the biopsied muscle tissue. Although no definitive diagnosis was reached at that time, a mitochondrial disorder was still favored and she was started on L-carnitine, creatine, and CoQ10 for that reason.

At initial evaluation with us, approximately 15 years after symptom onset, her exam revealed normal fundoscopic evaluation, normal pupils that were bilaterally reactive to light, symmetric and severe impairment of extraocular motility in all directions of gaze without misalignment, and moderate bilateral ptosis despite past surgical corrections. She did not have facial weakness, her hearing was normal to conversation, and the rest of the cranial nerves were intact. Her muscle strength, bulk, and tone were normal. There were no myotonia or fasciculations. Deep tendon reflexes were 2+ at biceps, brachioradialis, and triceps, and were bilaterally absent at patella and ankles. Plantar responses were flexor. Temperature and pin sensations were severely reduced in feet and hands, proprioception was impaired at great toes, the vibratory sensation was abolished at great toes and malleoli, and Romberg test was positive. There was no dysmetria on finger-to-nose or heel-to-shin tests. She had a mild wide-based gait; she was unable to walk in tandem but able to walk on heels and toes.

An autosomal recessive CPEO plus syndrome, involving the peripheral nerve, was then suspected. The coexistence of polyneuropathy prompted us to investigate nuclear DNA defects that might be causing secondary defects of mtDNA despite no alterations in mtDNA sequencing from the biopsied muscle, which might be explained by heteroplasmy, sampling bias, or lack of significant involvement of skeletal muscle in her case. More specifically, the presence of neuropathic pain pointed to consider a *POLG*-related disease as a possibility; whereas most “mitochondrial neuropathies” are painless, neuropathic pain appears to be more common in patients who have *POLG* mutations (7, 8). Genetic testing of *POLG* gene revealed a known compound of heterozygous mutations: one that comprises two single nucleotides *in-cis* (c.752 C>T and c.1760 C>T, which lead to p.Thr251Ile and p.Pro587Leu, respectively) and another one *in-trans* (c.1399G>A, p.Ala467Thr); these findings confirmed a *POLG*-related disease as unifying diagnosis.

## Discussion

Here, we present a patient with painless, progressive, bilateral, adult-onset ptosis and ophthalmoparesis, who also developed symptoms suggestive of peripheral nerve (polyneuropathy and neuropathic pain) and skeletal muscle (myalgias) involvement. This multi-organ phenotype suggested a mitochondrial disorder. An elevated serum lactic acid at baseline increased the diagnostic suspicion for a mitochondrial disorder. Although the presence of occasional subsarcolemmal mitochondrial accumulation and scattered COX-negative fibers on muscle biopsy could be aging-related findings in her case, a manifestation of a mitochondrial disorder could not be ruled out. However, the sequencing of mtDNA from biopsied muscle tissue was normal. In an affected organ (such as skeletal muscle), one would expect a single mtDNA deletion or point mutations of mtDNA in primary mitochondrial disorders, and multiple mtDNA deletions or mtDNA depletion in secondary mitochondrial disorders due to nuclear genetic defects. We suspected that the normal result of mtDNA sequencing from biopsied muscle and the non-specific and mild pathological findings contributed to delay in diagnosis in this case. However, although sequencing of mtDNA may identify deletions and point mutations, it may miss mtDNA depletion (loss of mtDNA copies) that would require assessment of copy number which was not performed. It is also plausible that skeletal muscle was not affected enough to reveal abnormalities of mtDNA (low or no mutant copies of mtDNA to detect), or that muscle sampling missed mtDNA abnormalities, or a combination of both. Thus, normal mtDNA sequencing should not discourage clinicians from further investigating the possibility of a mitochondrial disorder. Furthermore, neuropathy is rare in patients with single large-scale mitochondrial deletions or mtDNA point mutations (4, 12). Similar symptoms in her brother pointed to an autosomal recessive inheritance in her case which increased our suspicion for mutations in a nuclear gene. Lastly, polyneuropathy is a predictor of nuclear genetic defects in mitochondrial disorders, and the presence of neuropathic pain pointed to *POLG* gene as responsible for her phenotype (although most neuropathies associated with mitochondrial disorders are painless, painful neuropathies have been estimated to occur in up to a third of patients with *POLG* mutations) (7–9).

**Figure 2:**
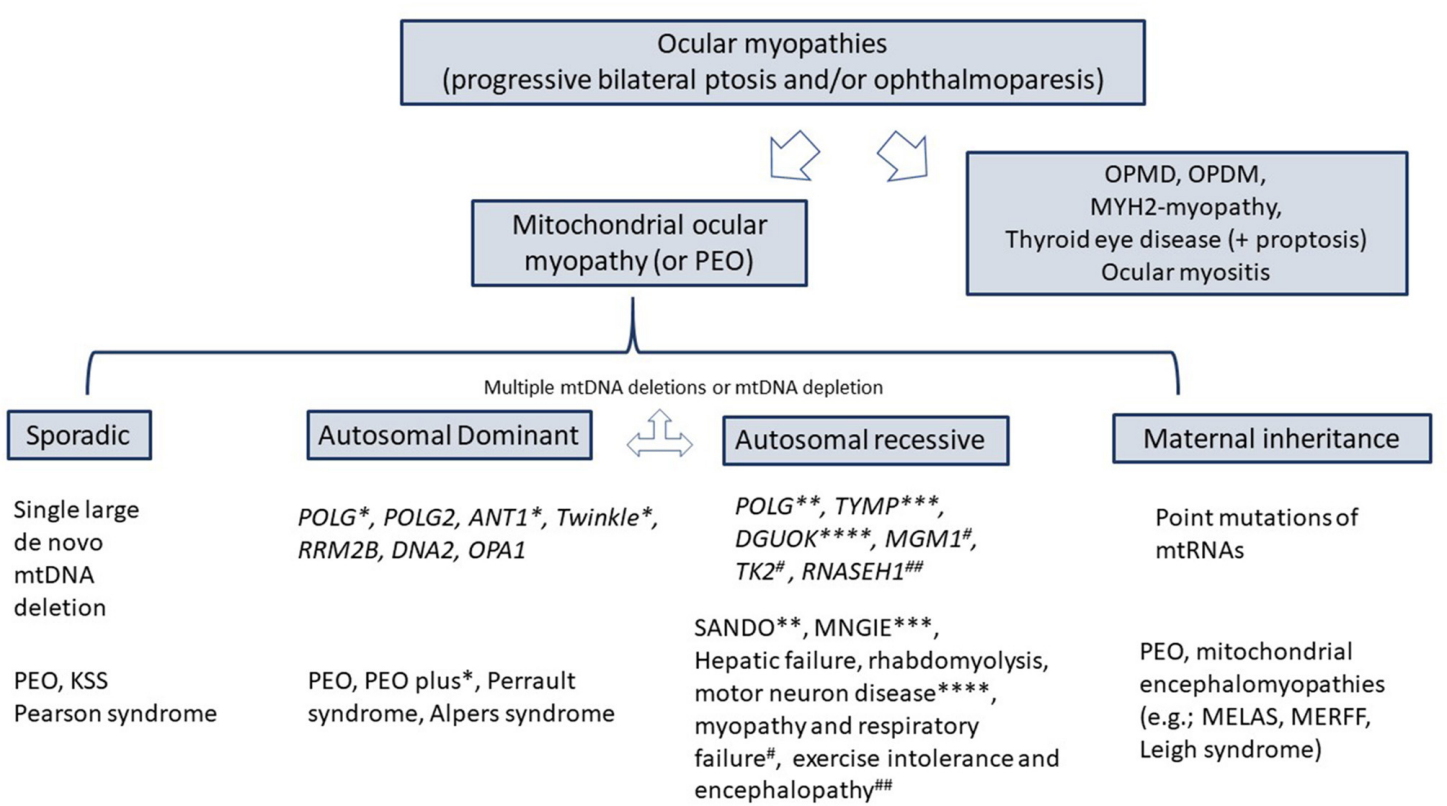
Differential diagnosis of ocular myopathies. KSS, Kearns-Sayre syndrome; SANDO, sensory ataxic neuropathy, dysarthria, ophthalmoplegia; MNGIE, Myo-neuro-gastrointestinal encephalopathy; OPMD, oculopharyngeal muscular dystrophy; OPDM, oculopharyngeal distal myopathy; MELAS, mitochondrial encephalomyopathy, lactic acidosis, and stroke-like episodes; MERRF, myoclonic epilepsy associated with ragged red fibers; POLG, Polymerase gamma; TYMP, thymidine phosphorylase.

The term CPEO (or PEO) usually applies to mitochondrial ocular myopathy; either due to mutations in mtDNA or nuclear DNA. However, ptosis, with or without ophthalmoparesis of similar characteristics as CPEO, can be seen in non-mitochondrial myopathies, such as oculopharyngeal muscular dystrophy, oculopharyngeal distal myopathy, or MHY2 myopathy (Figure 2). It is well known that the phenotypic spectrum of POLG-related disease is broad, and that awareness of such is key to diagnosing this disorder. In 2001, Van Goethem et al. reported the first *POLG* mutations as the cause of the CPEO phenotype (10). The number of *POLG* mutations and their associated clinical manifestations have dramatically expanded over the years (1). Identified *POLG* mutations in this patient (the complex p.Thr251Ile and p.Pro587Leu on one allele and p.Ala467Thr on the second allele) were previously reported in patients with CPEO or CPEO plus syndromes, and with infantile hepatocerebral syndromes (Alpers syndrome) (5, 11).

This patient underwent electrodiagnostic studies twice and a muscle biopsy. An axonal or mixed sensory polyneuropathy has been reported in patients with *POLG* mutations, but this electrical finding is not specific (12). Table 1 summarizes the electrical pattern of polyneuropathies in mitochondrial disorders in which CPEO is a hallmark. Likewise, and in addition to mild mitochondrial pathology in muscle fibers on muscle biopsy, the presence of neurogenic changes (angulated fibers, nuclear bags, and fiber type grouping) is also common in mitochondrial disorders but with no specific, and her history of lumbosacral radiculopathy may have also accounted for them (13).

**Table 1:** Electrodiagnostic features of “mitochondrial neuropathies” associated with CPEO.

	**Syndrome/disease**	**Nerve conduction studies**
**1. Mutations in nuclear genes**		
*POLG*	POLG-related disease	Axonal/mixed, mainly sensory PN
	SANDO	
*TYMP*	MNGIE	Demyelinating/mixed sensory-motor PN
**2. Mutations in either nuclear or mitochondrial genes**	Leigh syndrome	Demyelinating sensory-motor PN
**3. Point mutations of mtDNA**	MELAS/MERRF	
		PN not a main feature of phenotype
**4. Single large-scale deletion of mtDNA**	Kearns-Sayre syndrome	

*PN, polyneuropathy*.

Although polyneuropathy and neuropathic pain are common, a mitochondrial disorder should be considered in the presence of CPEO. Genetic testing in blood samples looking for nuclear defects should be the first step in this scenario. We would like to emphasize the importance of continuing to define and recognize clinical mitochondrial syndromes to avoid unnecessary testing and delays in diagnosis.

## Data Availability Statement

The original contributions presented in the study are included in the article/supplementary material, further inquiries can be directed to the corresponding author/s.

## Ethics Statement

Ethical review and approval was not required for the study on human participants in accordance with the local legislation and institutional requirements. The patients/participants provided their written informed consent to participate in this study. Written informed consent was obtained from the individual(s) for the publication of any potentially identifiable images or data included in this article.

## Author Contributions

ML-O: drafting and preparation of pathological studies (muscle biopsy). PG-P: design and drafting of the manuscript until final revision. Both authors contributed to the article and approved the submitted version.

## Funding

PG-P is funded by Muscle Study Group in collaboration with American Academy of Neurology and American Brain Foundation.

## Conflict of Interest

The authors declare that the research was conducted in the absence of any commercial or financial relationships that could be construed as a potential conflict of interest.

## Publisher's Note

All claims expressed in this article are solely those of the authors and do not necessarily represent those of their affiliated organizations, or those of the publisher, the editors and the reviewers. Any product that may be evaluated in this article, or claim that may be made by its manufacturer, is not guaranteed or endorsed by the publisher.
